# Raising the Alarm: Underdiagnosis of Cardiac Sarcoidosis in Young Patients With Unexplained Heart Block

**DOI:** 10.1016/j.jacadv.2025.101890

**Published:** 2025-06-20

**Authors:** Ghazaleh Goldar, Aaron A. Sifuentes, Kaushik Gokul, Usamah M. ElBakkush, Mohammed Mhanna, Peter Farjo, Paari Dominic

**Affiliations:** a Division of Cardiovascular Medicine, University of Iowa Hospitals and Clinics, Iowa City, Iowa, USA; b Saint Louis University School of Medicine, St. Louis, Missouri, USA; c Department of Internal Medicine, University of Iowa Hospitals and Clinics, Iowa City, Iowa, USA

**Keywords:** biopsy, cardiac MRI, cardiac sarcoidosis, complete heart block, FDG-PET

## Abstract

**Background:**

Cardiac abnormalities are often the first or only sign of sarcoidosis. Current guidelines recommend evaluating for cardiac sarcoidosis (CS) in patients below 60 years with unexplained complete heart block (CHB).

**Objectives:**

The aim of the study was to assess the proportion of patients with unexplained CHB who receive guideline-recommended testing for CS and to compare subsequent diagnosis rates with expected prevalence.

**Methods:**

We conducted a retrospective cohort study using TriNetX data, identifying patients aged 18 to 60 years with unexplained CHB requiring device placement over the past 5 years. We assessed the use of cardiac diagnostic tests—cardiac magnetic resonance imaging, positron emission tomography, chest computed tomography, and myocardial biopsy—and tracked CS diagnoses over 5 years.

**Results:**

Among 1,279 patients meeting criteria across 55 health care organizations, 75% were treated at academic centers. The mean age was 48 ± 11 years; 53% were male, and 73% were White. Over a median follow-up of 724 days (Q1-Q3: 0-1,117), advanced cardiac testing was performed in 256 patients (20.0%; 95% CI: 17.8%-22.2%). Specific test utilization included cardiac magnetic resonance imaging in 131 patients (10.2%; 95% CI: 8.6%-11.9%), chest computed tomography in 141 (11.0%; 95% CI: 9.3%-12.7%), cardiac positron emission tomography in ≤10 (0.7%; 95% CI: 0.3%-1.3%), and myocardial biopsy in ≤10 (0.7%; 95% CI: 0.3%-1.3%). Fewer than 10 patients (<1%; 95% CI: 0.3%-1.3%) were diagnosed with CS, far below the expected prevalence of 19% to 34%.

**Conclusions:**

Despite guideline recommendations, diagnostic testing for CS in younger patients with unexplained CHB remains low. This gap likely contributes to significant underdiagnosis and highlights the need for improved adherence to evaluation protocols.

Sarcoidosis is a disorder identified by granulomatous formation that can occur in any organ system and typically manifests in patients under the age of 50 years.^1^^,^^2^ Though lung involvement is the most prevalent, cardiac magnetic resonance imaging (cMRI) studies have demonstrated cardiac involvement meeting Heart Rhythm Society criteria in almost 1 of 3 patients and almost 40% of symptomatic patients.^3^^,^^4^ Initial clinical manifestations of cardiac sarcoidosis (CS) include symptomatic high-grade AV block, ventricular arrhythmias, heart failure, and sudden cardiac death.^5^^,^^6^

Perspectives **COMPETENCY IN MEDICAL KNOWLEDGE 1:** This study reveals a significant gap in care for patients with unexplained complete heart block, with many cases of CS likely remaining undiagnosed and untreated. Current guidelines recommend evaluating unexplained complete heart block for infiltrative disorders like CS using positron emission tomography imaging, cardiac magnetic resonance imaging, extracardiac sarcoid assessment, and/or myocardial biopsy.

**COMPETENCY IN MEDICAL KNOWLEDGE 2:** This study reveals a significant gap in care for patients with unexplained complete heart block, with many cases of CS likely remaining undiagnosed and untreated. It highlights the need for clinicians to consider CS as a potential etiology in patients below 60 years of age with unexplained heart block, which could influence device selection and patient outcomes.

**TRANSLATIONAL OUTLOOK:** To our knowledge, this is the first large-scale study across multiple health care organizations to assess how frequently practitioners follow guideline-recommended evaluation for CS in young patients aged 18 to 60 years who present with complete heart block and the subsequent diagnostic rate of sarcoidosis in these patients within 5 years.

When presenting with complete heart block (CHB), the timely diagnosis of CS is crucial, as an implantable cardioverter defibrillator may be recommended over a pacemaker.^7^ While data on the number of patients with undiagnosed CS after presenting with unexplained atrioventricular (AV) block are limited, smaller studies have indicated that this figure may be as high as 30%.^8^^,^^9^ These studies, along with the American Heart Association, recommend screening for CS in unexplained heart block patients under the age of 55 to 60 years.^8^, ^9^, ^10^

Diagnostic steps include performing a cMRI and evaluating for extracardiac sarcoidosis if the cMRI is positive.^10^ If the cMRI results are inconclusive, a fludeoxyglucose-18 positron emission tomography (PET) is recommended.^10^ The American Heart Association guidelines also suggest considering an endomyocardial biopsy if no evidence of extracardiac sarcoidosis is found.^10^ These diagnostic steps are crucial, as identifying CS can significantly impact the management approach and may influence the type of device used for treatment.

Because the proportion of patients with unexplained CHB who undergo guideline-recommended workup for CS remains unclear, we performed the first large-scale study across multiple health care organizations (HCOs) aiming to determine the percentage of patients aged 18 to 60 years with unexplained CHB who receive the necessary diagnostic evaluation for CS within 5 years, in accordance with established guidelines. By utilizing the TriNetX research network, we aimed to better understand this gap in clinical practice, which is crucial for early CS identification, appropriate management, and improving patient outcomes.

## Methods

### Study design

This was a retrospective observational study seeking to identify the number of patients presenting with unexplained CHB who received guideline-recommended workup of CS. A flowchart of this study's design can be found in the Central Illustration.

**Central Illustration:**
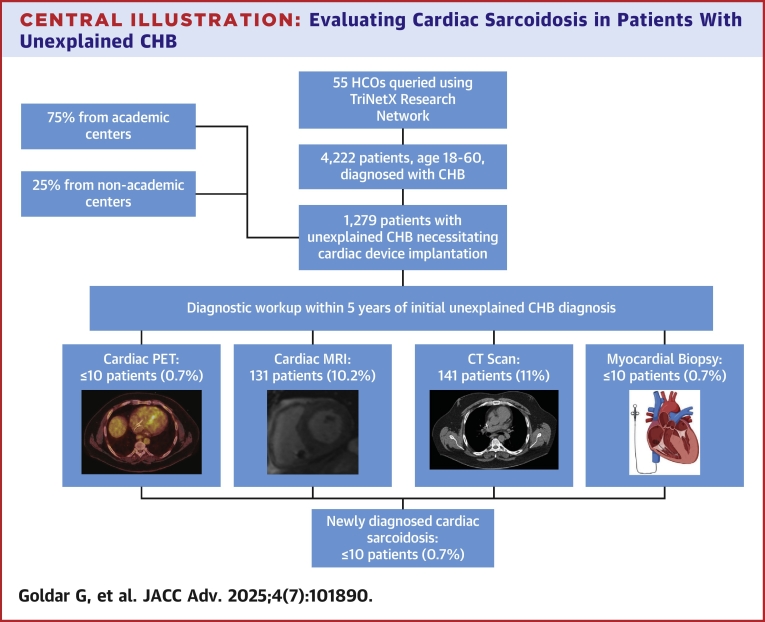
Evaluating Cardiac Sarcoidosis in Patients With Unexplained CHB Summary of the study design and key findings, highlighting the number of academic vs nonacademic centers involved, diagnostic testing rates, and the overall number of patients diagnosed with cardiac sarcoidosis. CHB = complete heart block; CT = computed tomography; HCO = health care organization; MRI = magnetic resonance imaging; PET = positron emission tomography.

### Data source

This study utilized data from the TriNetX research network (TriNetX, Inc). TriNetX is a global federated health research network providing access to electronic medical records (diagnoses, procedures, medications, laboratory values, and genomic information) across large HCOs. As a federated network, TriNetX received a waiver from Western Institutional Review Board since only aggregated counts and statistical summaries of deidentified information are received, no protected health information is received, and no study-specific activities are performed in retrospective analyses. This query was run in May 2025 on the network Research (with NLP) with 55 HCO(s).

### Patient selection

We utilized the International Classification of Diseases, Tenth Revision diagnosis and procedure codes, and the Centers for Medicare and Medicaid Current Procedural Terminology codes (Supplemental Table 1) to identify patients. We included patients between the ages of 18 and 60 years who had complete atrioventricular block necessitating placement of a cardiac implantable device within a month of diagnosis. Exclusion criteria broadly included patients with a prior diagnosis of sarcoidosis and those with CHB attributable to procedural or ischemic causes, infections, congenital anomalies, or prior chest radiation. A detailed list of inclusion and exclusion criteria can be found in Table 1.

**Table 1:** Inclusion and Exclusion Criteria

Inclusion criteria
Complete atrioventricular block followed by placement of an implantable cardiac device within 1 mo of diagnosis
Exclusion criteria
Previously diagnosed sarcoidosis
Hemochromatosis
Current heart transplant status
Ischemic heart disease
STEMI
Systemic connective tissue disorders
Endocarditis
Acute myocarditis
Procedures on the aortic valve, surgical or percutaneous
Myomectomy
Radiation to chest
Septal myectomy
Alcohol septal ablation
Congenital heart block
Positive Lyme Western blot

STEMI = ST-elevation myocardial infarction.

### Index event

The index event is defined as the time point at which the analysis begins. In our study, the index event was defined as the date on which a patient was diagnosed with CHB.

### Time window

Our analysis started on the first day of the index event and continued until 5 years after the first occurrence of the index event. Our cohort includes patients between the years of 2020 and 2025.

### Outcomes

The primary outcome of this study was the occurrence of either cardiac PET, cMRI, myocardial biopsy, or a diagnosis of sarcoidosis within 5 years following the diagnosis of CHB. Baseline demographics and outcomes were expressed as rates or percentages.

## Results

Between 2020 and 2025, a total of 4,222 patients aged 18 to 60 years were diagnosed with CHB. Among them, 1,279 patients with unexplained CHB met our inclusion criteria across 55 HCOs. Of this cohort, 956 patients (75%) were from academic centers (37 organizations), 298 (25%) from nonacademic centers (17 organizations), and the remainder from organizations with unknown classifications.

Baseline clinical characteristics and geographical details of the cohort are summarized in Tables 2 and 3, respectively. The mean age was 48 ± 11 years, 53% were male, 73% were White, and 10% were Black. Over a median follow-up of 724 days (Q1-Q3: 0-1,117), advanced cardiac diagnostic testing was performed in a subset of patients: cMRI in 131 patients (10.2%; 95% CI: 8.6%-11.9%), cardiac PET in ≤10 patients (0.7%; 95% CI: 0.3%-1.3%), and myocardial biopsy in ≤10 patients (0.7%; 95% CI: 0.3%-1.3%). Given that access to cMRI and PET may be limited at some institutions, we also examined how many patients underwent chest computed tomography (CT) to assess for possible evidence of sarcoidosis. We found that 141 patients (11%; 95% CI: 9.3%-12.7%) had some form of chest CT during the study period. Overall, 256 patients (20%; 95% CI: 17.8%-22.2%) underwent at least one of these evaluations (Table 4). Within the study period, <10 patients (<1%; 95% CI: 0.3%-1.3%) were diagnosed with CS. Characteristics and geographic distribution of patients who did vs did not undergo any diagnostic evaluation are detailed in Table 5.

**Table 2:** Baseline Characteristics of the Cohort (N = 1,279)

Age (y)	48 ± 11
Sex	
Male	678 (53%)
Female	562 (44%)
Ethnicity	
White	933 (73%)
Black	127 (10%)
Asian	38 (3%)
American Indian or Alaska	11 (0.9%)
Native Hawaiian or Other Pacific Islander	11 (0.9%)
Other or unknown race	153 (12%)
Comorbid conditions	
Hypertension	665 (52%)
Atrial arrhythmias	434 (34%)
Cardiomyopathy	383 (30%)
Diabetes	268 (21%)
Ventricular tachycardia	294 (23%)
Chronic kidney disease	140 (11%)

**Table 3:** Overall Geographic Distribution (U.S. Region) of Patients in the Initial Cohort (N = 1,279)

Northeast	383 (30%)
Midwest	204 (16%)
South	486 (38%)
West	166 (13%)
Unknown	38 (3%)

**Table 4:** Summary of Prevalence of Outcome Over 5-Year Period (N = 1,279)

cMRI	131 (10.2%; 95% CI: 8.6%-11.9%)
Cardiac PET	≤10 (0.7%; 95% CI: 0.3%-1.3%)
Chest CT	141 (11%; 95% CI: 9.3%-12.7%)
Myocardial biopsy	≤10 (0.7%; 95% CI: 0.3%-1.3%)
Patient with at least one of the following: cMRI, cardiac PET, myocardial biopsy, CT chest	256 (20%; 95% CI: 17.8%-22.2%)
Diagnosis of sarcoidosis	≤10 (<1%; 95% CI: 0.3%-1.3%)

cMRI = cardiac magnetic resonance imaging; CT = computed tomography; PET = positron emission tomography.

**Table 5:** Characteristics and Geographic Distribution of Patients Who Did vs Did Not Undergo Any Diagnostic Evaluation

	Patients With at Least 1 Diagnostic Testing (n = 256)	Patients Without Any Diagnostic Evaluation (n = 1,023)
Age (y)	48 ± 10	47 ± 11
Sex		
Male	133 (52%)	532 (52%)
Female	115 (45%)	460 (45%)
Ethnicity		
White	174 (68%)	767 (72%)
Black	33 (13%)	102 (10%)
Asian	10 (4%)	51 (5%)
American Indian or Alaska	0%	10 (1%)
Native Hawaiian or Other Pacific Islander	10 (4%)	10 (1%)
Other or unknown race	28 (11%)	1,112 (11%)
U.S. region		
Northeast	81 (31%)	287 (25%)
Midwest	39 (15%)	222 (20%)
South	90 (34%)	415 (37%)
West	44 (17%)	178 (16%)
Unknown	2 (<1%)	21 (<1%)
Comorbid conditions		
Hypertension	161 (63%)	660 (64%)
Cerebrovascular disease	34 (13%)	121 (11%)
Heart failure	95 (37%)	393 (38%)
Atrial arrhythmias	74 (29%)	376 (36%)
Ventricular tachycardia	52 (20%)	239 (21%)
Diabetes	58 (23%)	240 (23%)
Chronic kidney disease	27 (11%)	111 (10%)

## Discussion

This study aimed to examine the proportion of patients aged 18 to 60 years who presented with unexplained CHB requiring an implantable cardiac device and received the guideline-recommended diagnostic workup for CS. Our results show that very few patients underwent further evaluation for CS. Specifically, only 256 patients out of the 1,279 (20%) had PET imaging, cMRI, chest CT, or a myocardial biopsy following their presentation with CHB. Ultimately, around 1% of patients were diagnosed with sarcoidosis, a rate much lower than the reported prevalence of CS in patients with unexplained CHB, particularly in those aged <60 years.

While clinically evident CS occurs in <10% of patients with sarcoidosis, autopsies, histopathological examinations of explanted hearts, and multimodality cardiac imaging studies have revealed a higher prevalence, approaching 30%.^11^, ^12^, ^13^, ^14^ The low rate of CS diagnosis in our study, despite excluding several other potential causes of CHB, suggests there may be gaps in the evaluation process. This underdiagnosis may be influenced by several factors. The demands of a busy clinical practice can limit the ability to prioritize guideline-recommended diagnostic steps for evaluating CS. Moreover, the diagnostic workup often relies on advanced imaging modalities such as cardiac cMRI and PET, which may not be readily available at all institutions. To explore this further, we examined how many patients underwent chest CT—an imaging modality that is generally more accessible across hospitals—as an alternative means of evaluating for sarcoidosis. Even when including chest CT, only about 10% of patients underwent this imaging, highlighting a potential missed opportunity for evaluation.

Additionally, we recognize that it can be challenging to perform imaging soon after device placement due to limited arm mobility and potential device-related artifacts. Myocardial biopsy, being more invasive, is typically reserved for select cases and is often pursued only after initial evaluations, including imaging for extracardiac sarcoidosis, have been completed.^10^ Furthermore, challenges such as limited access to specialized centers, multidisciplinary teams, or delays in referrals may also play a role. A retrospective study evaluating patients referred to advanced heart failure centers found that delayed referrals were common and associated with poorer outcomes.^15^ These findings mirror the challenges in diagnosing CS, where delayed or missed evaluation can similarly impact treatment decisions and long-term outcomes. Our results reinforce the importance of increasing clinician awareness around appropriate diagnostic triggers and ensuring early referral to centers equipped to evaluate and manage suspected CS. Overall, while these barriers are not necessarily a reflection of a lack of awareness, they can contribute to the underdiagnosis of CS in patients presenting with unexplained CHB.

Undiagnosed CS can result in serious complications, including progression of atrioventricular block, ventricular arrhythmias, heart failure, and sudden cardiac death.^5^^,^^6^ Advanced imaging plays a critical role in these patients by providing essential information to guide clinical decision-making and risk stratification. It enables the evaluation of left ventricular ejection fraction (LVEF), identification of myocardial scarring, and detection of active disease requiring immediate treatment, all of which are pivotal for tailoring appropriate management strategies.^16^^,^^17^ This information is crucial for selecting the most appropriate implantable device and tailoring patient management strategies. Current expert guidelines recommend the implantation of an implantable cardioverter defibrillator in patients with CS and an LVEF <35%.^18^ However, some retrospective studies suggest that even a mild reduction in LVEF (around 42%) may serve as an optimal cutoff for predicting ventricular arrhythmias and all-cause mortality in CS patients.^19^

## Strengths

The use of the TriNetX research network provided access to a large, geographically diverse cohort of patients across 55 HCOs. This broad population enhances the generalizability of the study's findings. This study specifically targeted patients presenting with unexplained CHB, a clinical scenario where identifying underlying CS is crucial. By focusing on this subset of patients, the study fills an important gap in understanding the prevalence of guideline-recommended diagnostic workup for CS.

### Study Limitations

As a retrospective observational study, there may be inherent biases in how data were collected and interpreted, making it susceptible to selection bias. Additionally, the TriNetX database predominantly represents data from the United States, which may limit the generalizability of our findings to other populations or health care systems outside of this region. The study relies on the International Classification of Diseases-10th Revision and Current Procedural Terminology codes for patient identification and outcome assessment, which means the quality and completeness of the data are contingent upon the accuracy of the coding process. Inaccurate or incomplete coding could introduce potential bias or misclassification. Additionally, while we are able to distinguish between academic and nonacademic centers, unfortunately, the TriNetX platform does not provide sufficient detail to identify whether any of these institutions are designated sarcoidosis centers of excellence. Another important limitation of this study is that although fewer than 1% of patients in our cohort were diagnosed with CS, we are unable to determine how those diagnoses were made given that the TriNetX database does not provide detailed information on each individual patient to protect their privacy. Furthermore, the diagnostic criteria for CS remain variable in clinical practice, which likely contributes to underdiagnosis in this cohort and limits the interpretability of our findings related to this subgroup. Lastly, the database did not allow for outcome stratification of underserved populations, which would further offer insight into the gap in care.

## Conclusions

This study highlights a significant gap in the evaluation and diagnosis of CS in patients presenting with unexplained CHB. Despite the established guidelines recommending comprehensive diagnostic workups, including cMRI, PET imaging, and myocardial biopsy, only a small proportion of patients received these assessments. Additionally, because advanced imaging modalities such as cMRI and PET may not be readily available at all institutions, we also evaluated the use of chest CT—an imaging technique that is generally more accessible in hospital settings—as an alternative method for assessing potential sarcoidosis. Even with the inclusion of chest CT, only about 10% of patients underwent this imaging, underscoring a potential missed opportunity for diagnostic evaluation. This underdiagnosis poses a considerable risk for delayed intervention and worsened clinical outcomes, such as the progression of heart block, ventricular arrhythmias, heart failure, and sudden cardiac death.

The findings underscore the need for increased awareness among clinicians about the importance of timely and appropriate diagnostic testing for CS, particularly in younger patients with unexplained heart block. Additionally, access to advanced imaging techniques and multidisciplinary care teams is crucial to ensure accurate risk stratification and appropriate treatment decisions, including the choice of implantable device. Prompt referral to specialists when CS is suspected is also critical to avoid delays in care. Future studies are needed to explore the underlying barriers to guideline adherence and to develop strategies for improving the diagnosis and management of CS, ultimately optimizing patient care and outcomes in this high-risk population.

## Funding support and author disclosures

The authors have reported that they have no relationships relevant to the contents of this paper to disclose.
