# Supplementary material for: Raising the Alarm: Underdiagnosis of Cardiac Sarcoidosis in Young Patients With Unexplained Heart Block
Source: JACC Adv. 2025 Jun 20;4(7):101890. doi: 10.1016/j.jacadv.2025.101890 (PMC12221766; doi:10.1016/j.jacadv.2025.101890)
Supplement: Supplementary data [file mmc1.pdf]

Supplementary Index

Supplementary Table 1: List of ICD-10-CM and CPT codes. (Intended for publication)

Variable	ICD-10-CM or CPT Code(s)	
	**Diagnosis Code**	**Procedural Code**
**Inclusion Criteria**		
Complete Atrioventricular Block	UMLS:ICD10CM:I44.2	
Transcatheter insertion or replacement of permanent leadless pacemaker, right ventricular, including imaging guidance (eg, fluoroscopy, venous ultrasound, ventriculography, femoral venography) and device evaluation (eg, interrogation or programming), when performed		UMLS:CPT:33274
Insertion of new or replacement of permanent pacemaker with transvenous electrode(s); ventricular		UMLS:CPT:33207
Insertion of new or replacement of permanent pacemaker with		UMLS:CPT:33208

transvenous electrode(s); atrial and ventricular		
Insertion of pacing electrode, cardiac venous system, for left ventricular pacing, at time of insertion of implantable defibrillator or pacemaker pulse generator		UMLS:CPT:33225
Insertion of pacing electrode, cardiac venous system, for left ventricular pacing, at time of insertion of implantable defibrillator or pacemaker pulse generator		UMLS:CPT:33249
**Exclusion Criteria**		
Raised Antibody Titer for lyme disease	UMLS:ICD10CM:R76.0	
Hemochromatosis	UMLS:ICD10CM:E83.11	
Heart Transplant Status	UMLS:ICD10CM:Z94.1	
Ischemic Heart Diseases	UMLS:ICD10CM:I20-I25	
Systemic Connective Tissue Disorders	UMLS:ICD10CM:M30-M36	

Acute and Subacute Infective Endocarditis	UMLS:ICD10CM:I33.0	
Acute Myocarditis	UMLS:ICD10CM:I40	
Surgical Procedures on the Aortic Valve		UMLS:CPT:1006141
Myectomy		UMLS:SNOMED:36143002
Respiratory System / Other Radiation / Mediastinum		UMLS:ICD10PCS:DBY6
Septal myectomy		UMLS:SNOMED:717764002
Percutaneous transcatheter septal reduction therapy (eg, alcohol septal ablation) including temporary pacemaker insertion when performed		UMLS:CPT:93583
Congenital heart block	UMLS:ICD10CM:Q24.6	
ST elevation (STEMI) MI of unspecified site	UMLS:ICD10CM:I21.3	
Sarcoidosis	UMLS:ICD10CM:D86	
**Outcomes**		
PET		UMLS:CPT:78433<br>UMLS:CPT:78432
cMRI		UMLS:CPT:75561
Myocardial Biopsy		UMLS:CPT:93505

CT Scan of chest		UMLS:CPT:71250<br><br>UMLS:CPT:71260<br><br>UMLS:CPT:71270<br><br>UMLS:CPT:BW24<br><br>UMLS:CPT:BW25
Sarcoidosis	UMLS:ICD10CM:D86	
